# Diet-gut microbial interactions influence cancer immunotherapy

**DOI:** 10.3389/fonc.2023.1138362

**Published:** 2023-03-24

**Authors:** Xue Wang, Shitao Geng

**Affiliations:** ^1^ Department of Oncology, First People’s Hospital of Guangyuan, Guangyuan, China; ^2^ Department of Emergency, First Naval Hospital of Southern Theater Command, Zhanjiang, China

**Keywords:** diet, gut microbiota, immunity, cancer, immunotherapy

## Abstract

The gut microbiome is involved in the absorption and metabolism of host nutrients and modulates the immune response, affecting the efficacy of immunotherapy for cancer. In patients receiving immunotherapy, appropriate modifications of gut microbiota are thought to improve therapeutic response. Of all the factors that influence the gut microbiota, diet is the most influential and modifiable. Healthy dietary patterns as well as some specific dietary components can help the growth of beneficial microbiota in the gut, thereby protecting against cancers and promoting human health. A growing number of researches have confirmed the positive effects of a diet-gut microbiota approach as an adjuvant therapy for cancer, but controversy remains. Here, we summarize the interactions between diet and gut microbes based on previous studies, and discuss the role of gut microbiota-based dietary strategies in tumor immunotherapy, with the potential mechanisms of actions also intensively discussed.

## Introduction

The gut microbiota is a highly complex community, it has been described as a virtual organ owing to the myriad of functions it performs, including the production of bioactive metabolites, regulation of immunity, energy homeostasis and protection against pathogens (1). The crosstalk between the host and the gut microbiota generates an intestinal homeostasis beneficial to the host (2). Dysregulation of homeostasis has been implicated in diseases including cancer (3, 4). Meng et al. (5) summarized that gut microbial dysbiosis contributes to cancer susceptibility through multiple pathways, including causing chronic inflammation to promote tumor progression and accelerate invasion and metastasis, triggering innate and adaptive immune responses involved in tumor formation and generating metabolites to promote malignant transformation. Further studies have shown that the microbiota and its associated metabolites are not only closely associated with carcinogenesis by inducing inflammation and immune dysregulation, but also interfere with the efficacy of anticancer drugs (5).

The influence of gut microbiota on various physiological functions depends on the quantity and quality of microorganisms and its metabolic potential, which is determined by many factors (1). Diet, one of the most important influencers of gut microbiota homeostasis, has a major impact on the gut microbiota (6–9). Data indicate that specific nutrients, especially proteins and insoluble fiber, have profound effects on gut microbial community structure, function, and the secretion of metabolites that modulate immune function and multiple metabolic and inflammatory pathways (10, 11). Indeed, diet is also involved in cancer development and prevention. A recent analysis of two large prospective cohort studies showed a potential link between Western diet (WD), gut microbiota and colorectal carcinogenesis, with WD being associated with a higher incidence of pks+ E. coli-rich cancers (12). The World Cancer Research Fund/American Institute for Cancer Research (WCRF/AICR) report also states that the Western diet increases cancer risk and that cancer risk is higher in people on this type of diet. In contrast, consumption of whole grains, dietary fiber and dairy products reduces cancer risk and is associated with better anti-cancer treatment with better quality of life and increased gut microbial population and diversity (13–16). Diet, as a key determinant of microbiota structure, influences the whole gut microbiota mainly by modulating the abundance of specific species and their individual or collective functions (17, 18). Specific dietary patterns elicit different changes in the gut microbiota. How to use diet to regulate gut microbiota so as to affect the development, metastasis and treatment of tumors is one of the most concerned issues in recent years, which requires a deeper understanding of the interaction mechanism between diet and gut microbiota, but the current research on this aspect is insufficient. In this review, we discussed the interaction between diet and microbiota, how diet and microbiota crosstalk affect the occurrence and progression of tumors, and the application of diet and microbiota crosstalk in cancer immunotherapy.

## Dietary patterns influencing gut microbiota composition

Increasing evidence suggests that gut microbiota can influence the susceptibility and etiology of cancer (19). Diet can affect this process, it can promote the growth of bacteria with anti-tumor or carcinogenic properties, and can also be metabolized by intestinal bacteria into some bioactive ingredients to prevent cancer, depending on the nutrient composition (20). Population based studies have shown that diet is the main determinant of microbiota variation among individuals (17, 18). Changes in a large number of nutrients in the diet, including fat, protein and carbohydrate, lead to significant changes in human intestinal microbiota (21, 22). The difference of nutrient composition in different dietary patterns results in different intestinal microbial results. Several popular diets, including western diet (WD), Mediterranean diet (MD), ketogenic diet (KD), have been studied for their ability to modulate the intestinal microbiota (**Table 1**) (23), thus affecting host metabolism and immunity.

**Table 1:** Effects of different dietary patterns on the gut microbiota.

Dietary patterns	Total effects	Specific	
Western diet	Decreasing microbiota diversity	↑*Bacteroidetes* ↑*Alistipes* ↑*Bilophila* ↑*Ruminococcus torques* ↑*Bacteroides* spp. ↑*Akkermansia*	↓*Proteobacteria* ↓*Firmicute* ↓*Prevotella* ↓*Roseburia* ↓*Eubacterium rectale* ↓*Ruminococcus bromii*
ketogenic diet	Beneficial bacteria increased and pathogenic bacteria decreased	↑*Bacteroidetes* ↑*Bacteroides* ↑*Bifidobacterium* ↑*Prevotella* ↑*Desulfovibrio* spp. ↑*E. coli* ↑*Akkermansia*	↓*Bifidobacteria* ↓*Proteobacteria* ↓*Cronobacter* ↓*E. rectale* ↓*Dialister*
Mediterranean diet	Increasing microbiota diversity	↑*Bacteroides* ↑*Bifidobacteria* ↑*Bacteroidetes* ↑*Clostridia* ↑*Lactobacillus* ↑*Prevotella* ↑*Eubacteria*	↓*Enterobacteria* ↓*Proteobacteria* ↓*Firmicutes*
Vegetarian Diets	Indeterminism	↑*Prevotella/Bacteroides* ↑*Bacteroides* ↑*Clostridium* *clostridioforme* ↑ *Klebsiella pneumoniae*	↓ *Clostridium* cluster XIVa ↓*Bilophila wadsworthia* ↓*Bifidobacteria* ↓*C. perfringens*
Vegetarian Diets		↑*Faecalibacterium* ↑*Bifidobacterium* ↑*Lactobacillus* ↑*E. rectale*	↓*C. histolyticum*
high-glucose diet (HGD)	Decreasing microbiota diversity	↑Proteobacteria ↑*Desulfovibrio vulgaris* ↑Firmicutes/Bacteroidetes	↓Bacteroidetes
Low-FODMAP Diet	Reduction total bacterial abundance		↓*Clostridium* cluster IV ↓*Propionibacteriaceae* ↓*Akkermansia muciniphila* ↓*Ruminococcus gnavus* ↓*Bifidobacteria*
gluten-free diet (GFD)	Decreasing microbiota diversity (healthy bacteria decreased, unhealthy bacteria increased)	↑*Enterobacteriaceae (E. coli*) ↑*Victivallaceae* ↑*Clostridiaceae* ↑*Coriobacteriaceae*	↓*Bifidobacterium* ↓*Clostridium lituseburense* ↓*Faecalibacterium prausnitzii* ↓*Ruminicoccus bromii* ↓*Roseburia feces* ↓*B. longum* ↓*Lactobacillus*

**Figure 1:**
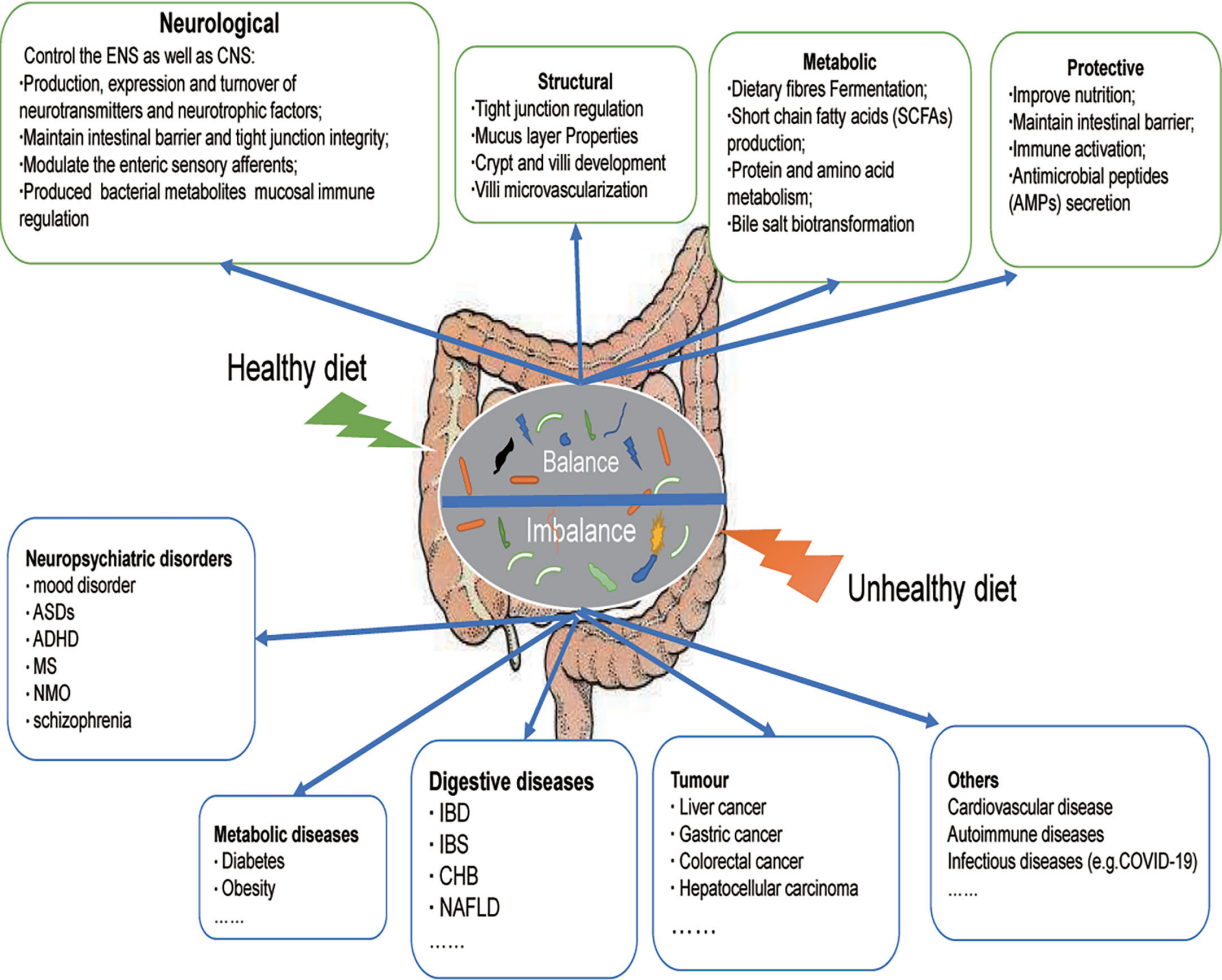
Dietary modulation of the gut microbiota. A healthy diet can maintain the balance of intestinal microecology, and is beneficial for the gut microbiota to exert positive effects on the host (neurological, structural, metabolic, protective). An unhealthy diet disrupts the balance of the host gut microecology and participates in the occurrence and development of many diseases, including neuropsychiatric disorders, metabolic diseases, digestive diseases, tumor, and others. ASDs, Autistic Spectrum Disorder; ADHD, Attention deficit and hyperactivity disorder; MS, Multiple Sclerosis; NMO, neuromyelitis optica; IBD, Inflammatory bowel disease, IBS, Irritable Bowel Syndrome; CHB, Chronic hepatitis B; NAFLD, Nonalcoholic fatty liver disease.

Unhealthy dietary patterns can cause gut microbial imbalance, which is related to many diseases (**Figure 1**). WD is the most common pattern, a diet rich in sugar, salt and/or fat, has been widely recognized as a major factor in the onset of metabolic disorders and associated pathological conditions (24). It can have deleterious effects on the intestinal barrier, leading to leaky gut, gut microbial dysbiosis, and altered metabolites, further contributing to local inflammation and the presence of Lipopolysaccharide (LPS) in the bloodstream, which can lead to systemic endotoxemia and chronic inflammation (24). The WD has a profound impact on the diversity and populations of gut microbial species (25), which is characterized by a significant decrease in intestinal microbial diversity and an increase in the proportion of harmful bacteria. Animal models have shown that WD patterns result in decreased levels of *Bacteroidetes* and increased levels of both *Proteobacteria* and *Firmicute*(26). A significant increase in the abundance of *Proteobacteria*, *Bilophila wadsworthia*, was also reported in another study (27). A recent cross-sectional and longitudinal study also confirmed that long-term western dietary patterns can reduce the diversity and function of intestinal microorganisms (28). With the increase of western dietary time, bacterial strains from *Prevotella* are replaced by dominant strains from *Bacteroides*, and the proportion of *Bacteroides* to *Prevotella* increases 10 times.

There are studies showing that people on a WD pattern exhibit a considerably higher *Firmicutes : Bacteroidetes* ratio, less antimicrobial paneth activity, higher concentrations of proinflammatory cytokines, including interferon (IFN)-γ, tumor necrosis factor (TNF)-α, interleukins (IL)-1β and IL-6 (29), and bacterial translocation leading to endotoxemia (30). These changes will lead to an increased risk of many cancers (31) and affect the treatment of tumors (32). For example, a low fiber, high-fat diet can lead to *F. nucleatum* levels increased significantly (33), which had a strong correlation with colorectal cancer (34). *F. nucleatum* can activate E-cadherin/β-catenin signal transduction promotes colorectal carcinogenesis and enhances tumor immune evasion by creating a proinflammatory microenvironment conducive to colorectal cancer development through recruitment of immunosuppressive myeloid cells and suppression of natural killer (NK) cell function (35–37). Meanwhile, it is also associated with a lower density of tumor infiltrating CD3^+^T cells (38), thereby limiting the role of immunotherapy for colorectal cancer, which may be one of the breakthroughs to enhance the efficacy of immunotherapy in the future.

A healthy dietary pattern benefits the stabilization of the intestinal internal environment and increases intestinal microbial diversity (**Figure 1**), including MD and KD. The MD consists mainly of cereals, nuts, legumes, vegetables, and fruits, with a moderate consumption of poultry and fish (39). The MD is now a popular healthy eating pattern that not only improves overall health, but also reduces the risk of non-communicable diseases (NCDs) (40, 41). MD-based nutritional intervention trials have clarified their possible mechanisms in the host, including the following: reduction of serum lipid levels; prevention of oxidative stress, inflammation and platelet aggregation; regulation of hormones and growth factors associated with cancer development; amino acid restriction-induced inhibition of nutrient-sensing pathways; and regulation of microbial metabolism to promote the normal function of host metabolism(42). Of these, effects on microbes are considered to be key factors in the prevention of NCDs, including cancer.

Studies have shown that the intake of a MD can significantly reduce the incidence of many diseases, such as neurodegenerative diseases (43), cancer (44), obesity (45), type 2 diabetes (46), inflammatory diseases (47) and cardiovascular disease (CVD) (48). This may be mediated by the anti-inflammatory potential of the MD while also being associated with unique gut microbiota alterations caused by the MD (49), namely increased abundances of *Bacteroidetes* and *Clostridia*, and decreased abundances of *Proteobacteria* and *Firmicutes* (47, 50). This alteration in the gut microbiota favors the production of high levels of short chain fatty acids (SCFAs), including acetate, propionate and butyrate, which can suppress the development of inflammatory, autoimmune, and allergic diseases (51).

A clinical trial (NCT02118857) (50) has shown a significant association between MD and increased levels of fecal SCFAs, *Prevotella* and some fiber-degrading *Firmicutes*, and consumption of MD has been associated with a beneficial microbiome related metabolic profile. This microbial signature elicited by MD has also been suggested to have an effect on prevent cancer (41), and interestingly this effect is not restricted to the gut but also affects cancer risk in organs outside the gut by modulating the local microbiota (52, 53). Data from the EPIC study (52) suggest that MD represents the best food model for cancer prevention and is associated with a reduced risk of gastrointestinal cancers, and data from the MOLI-SANI study (54) found that the MD model reduced levels of inflammatory markers such as C-reactive protein (CRP), white blood cells, platelet count and granulocyte/lymphocyte ratio, the latter being associated with poorer cancer prognosis and is an independent predictor of tumour growth, progression and metastatic processes (55). Recently, Shivley et al. (53) showed that MD intake resulted in a 10-fold increase in the abundance of mammary *Lactobacilli* (negative regulators of breast cancer) compared to the WD, accompanied by increased levels of mammary bile acid metabolites and reduced reactive oxygen metabolites. In conclusion, the MD pattern seems to be associated with features related to the beneficial microbiota inside and outside the gut, and more studies are needed to seek the specific role of its effect on the gut microbiota.

The KD, a high-fat, low carbohydrate diet (56), may promote metabolic health and the prevention of cancer and other NCDs through a variety of mechanisms, including (1) reduced insulin levels; (2) enhanced oxidation of mitochondrial substrates leading to sustained mild elevation of mitochondrial reactive oxygen species production and antioxidant adaptation; and (3) specific antioxidant and anti-inflammatory effects of the ketone body β-hydroxybutyrate as histone deacetylase (HDAC) inhibitors (57). In cancer, lowering circulating glucose levels and inducing ketosis leaves cancer cells lacking energy while normal cells adapt their metabolism to use ketone bodies and survive. In addition, by lowering glucose, levels of insulin and insulin-like growth factor, which are important drivers of cancer cell proliferation, are reduced. In most preclinical tumour models, KDs have been shown to inhibit glycolysis and proliferation of cancer cells(58, 59) and may be used as an adjuvant therapy for cancer (58).

One of the key players in the mechanism of action of the KD may be the gut microbiota. But its effect on the gut microbiota is poorly understood and only limited studies have shown a link between the KD and the gut microbiota. The possible effects of KD on gut microbes have been summarized in reviews by Fan et al. (60) and Paoli et al. (61). However, the ultimate role of the microbiota in mediating the anti-tumour effects of KD has not been investigated to date. Overall, the main effects of the KD on the gut microbiota were as follows: the changes of intestinal microflora α-diversity and richness decreased, beneficial bacteria increased and pathogenic bacteria decreased, metabolic substances such as SCFA increased/decreased, hydrogen sulfide (H2S) increased and lactic acid decreased (61, 62).Indeed, KD have paradoxical effects on the gut microbiota, in a recent study, the fecal microbiological profile of individuals with GLUT1 deficiency syndrome after KD treatment showed significantly increased levels of *Desulfovibrio spp*, a group of bacteria thought to contribute to the exacerbation of intestinal mucosal inflammation caused by fat intake (63, 64). Therefore, KD may have different efficacy in altering the composition of the gut microbiota.

In addition, the gut microbiome also includes fungi and viruses. The gut viral community includes eukaryotic viruses and phages (65), which can be influenced by diet and have the potential to affect host function by interacting with gut bacteria and/or altering gene expression in the host (66). The study (67) has shown that malnutrition is associated with enterovirus-associated mortality. Diets deficient in nutrients (e.g., vitamin D, zinc) increase susceptibility to enteroviruses (68, 69). However, there is a lack of corresponding studies on how different dietary patterns affect enteroviruses. Similarly, many species of fungi have been detected in the human gut and are influenced by environmental factors (70). Nagpal R, et al. (71) found that a modified Mediterranean ketogenic diet (MMKD) has a broad effect on fungal diversity and modulates mycobacterial biomes related to fungal-bacterial co-regulatory networks. Previous study (72) also showed a direct association between diet and fungi, such as Methanobrevibacter and Candida were positively associated with a diet high in carbohydrates but negatively associated with a diet high in amino acids, proteins and fatty acids. Limited evidence suggests that Candida albicans may interact with immune cells in the host gut through pathways associated with Dectin-1, Toll-like receptor 2 (TLR2), and TLR4, and disrupt fungal-host-microbiota interactions (73). However, the relationship between diet and fungal communities (and function), and the underlying mechanisms remain unclear (74).

## Role of the gut microbiota in diet

Gut microbiota play an important role in human metabolism and health. The close interaction between gut microbiota and food intake not only helps to degrade food nutrients, but also can synthesize a variety of nutrients for human use (75).

Recently, a systematic review (76) of the effects of gut microbiota on nutrients and non-nutrients demonstrates that gut microbiota plays a key role in substrate metabolism, especially in the breakdown and transformation of the dietary substrates examined. Gut microbiota is related to energy homeostasis, it plays an important role in energy absorption, storage and consumption from diet. Gut microbiota interacts with the gut environment, breaking down undigested polysaccharides, producing monosaccharides and SCFAs, and allowing the host to recover energy from other undigested dietary matrices. Gut microbes also affect energy balance by regulating the metabolites produced by gene expression (77–81).

The absorption of nutrients mainly occurs in the duodenum and upper jejunum. The undigested dietary components reach the large intestine and are fermented by the intestinal anaerobic microbial community to produce a wide range of metabolites, which reflects the significant biochemical capacity of the microbial community (82, 83). The main fermentation products of healthy adults are gases and organic acids, especially three SCFAs: acetate, propionate and butyrate (84). SCFAs plays an important role in human body. In addition to providing nutrients to enterocytes, SCFAs act as signaling molecules that bind to G protein-coupled receptors (GPR) 41 and GPR43 on the surface of intestinal epithelial and immune cells, regulate the secretion of pro-inflammatory cytokines such as IL-18, and act on the central nervous system to regulate food intake and energy expenditure *via* glucagon-like peptide 1 (GLP1) and peptide tyrosine-tyrosine (PYY). In addition, SCFAs act as HDAC inhibitors in immune cells and adipocytes, regulating transcription in these cells through chromatin status (22). The production of SCFAs is produced by the fermentation of intestinal specific bacteria using nutrients that cannot be directly absorbed by the host in the diet. The change of bacterial composition will change the distribution of SCFAs in feces.

Early studies have shown that the colonic microbiota has a considerable ability to hydrolyze protein, transforming the dietary protein and endogenous protein from host enzyme, mucin and exfoliated intestinal cells into short peptides, amino acids and derivatives, short chain and branched fatty acids and gases, which is conducive to the rapid absorption of nutrients and the excretion of harmful substances (85). It was confirmed that *Bacteroides* and *Propionibacterium* were the main proteolytic bacteria in fecal samples (85). Dai et al. (86) also showed that intestinal bacteria can transform free amino acids into peptides, which contributes a lot to the metabolism and bioavailability of amino acids in mammalian intestine. Gut microorganisms can degrade undigested luminal proteins and peptides in amino acids. Although these amino acids cannot be absorbed by colon epithelium to a large extent, they are precursors of many end products of metabolism, such as SCFAs and organic acids (87), which affect the metabolism of the body. This indicates that intestinal microorganisms can not only absorb and degrade some amino acids, but also synthesize amino acids, suggesting that amino acid exchange between microbial population and host can be carried out in two directions, although it is not clear yet (87).

In addition, studies in the past 40 years have found that intestinal microbiota can synthesize some vitamins, especially vitamin K and B, including biotin, cobalamine, folate, niacin, pantothenic acid, pyridoxine, riboflavin and thiamine (88). And their vitamin synthesis pathway patterns complement each other (89), and there is cross feeding between intestinal microorganisms (90).In short, undigested carbohydrates and proteins in the diet can be fermented by intestinal bacteria to produce SCFAs and gases (CO2, CH4), which are further absorbed or excreted by the colon and account for 10% - 30% of the total food energy supply. Therefore, intestinal flora can enhance human intestinal digestion ability and regulation of energy balance play an important role. Intestinal bacteria rich in enzymes are also necessary for the synthesis of vitamins, and play an important role in the absorption of minerals (91). It can be seen that the reciprocal relationship with intestinal microorganisms may provide a variety of beneficial nutritional functions for the host, including the degradation and detoxification of dyspepsia food, as well as the synthesis of essential nutrients (92).

The role of the gut microbiota in nutrition can be broadly divided into two categories: one is the indirect effect on regulatory pathway, the other is the direct effect on digestion process and nutrition supply (93). Some bacterial species, such as *Lactobacillus plantarum* or *Acetobacter*, can promote growth by activating the insulin pathway, and partially compensate for the harmful effects of malnutrition (94, 95). In addition, gut microbes can directly promote the nutrient supply to the host and largely affect the metabolism of lipids and sugars (96). Further studies have shown that gut microbes can increase peptidase activity or provide secondary metabolites, vitamins and amino acids (97–99). On the other hand, the composition of gut microbiota is affected by diet. Different diets seem to have the most profound effect on the quality of the gut microbiota. A healthy diet is rich and comprehensive in nutrients, which helps to promote the diversity and functionality of the gut flora so that it can effectively serve its host (1).

**Figure 2:**
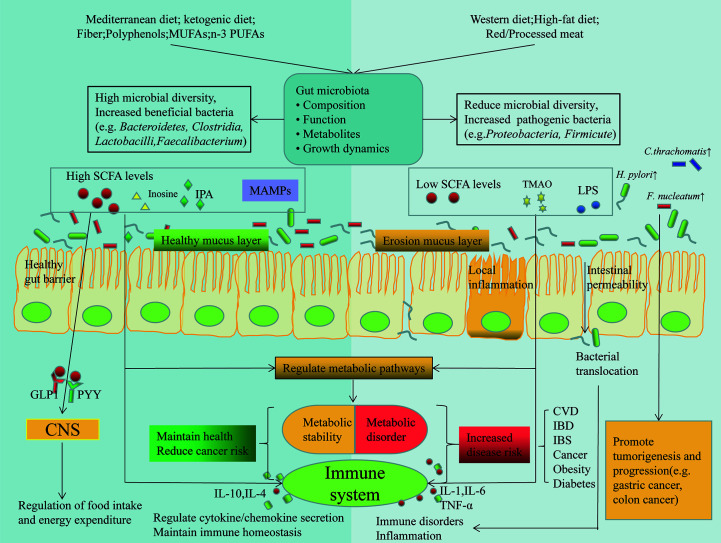
Dietary and gut microbial interactions regulate host immunity and metabolism. Different dietary patterns affect the composition of gut microbes and their metabolites, which are involved in the pathogenesis of many diseases by affecting host metabolism and immunity through different mechanisms. Also alterations in gut microbes in turn affect nutrition. Some tumor associated bacterascites (e.g. *H. pylori, F. nucleatum* and *C. thrachomatis*), promote tumorigenesis and progression. GLP1, glucagon-like peptide 1; PYY, peptide tyrosine-tyrosine; IPA, indolepropionic acid; MAMPs, Metabolism-Associated Molecular Patterns; TMAO, Trimetlylamine oxide.

In summary, there is an interaction between nutrients and the gut microbiota in ways that can be direct or indirect, by providing or not providing some of the dietary components required for microbial growth, or by affecting host metabolism and immune responses, or by disrupting the protective function of the gut barrier (**Figure 2**) (100).

## Dietary and gut microbial interactions in cancer immunotherapy

Gut microbes and diet tamper with each other to perform many functions in the host, including synthesis and regulation of bioactive compounds, host metabolic regulation and host immune regulation(22). Interactions between diet and gut microbiota may influence cancer development by altering host metabolism and the immune system (101), as well as shaping cancer immune surveillance and immunotherapeutic responses (102, 103).

**Figure 3:**
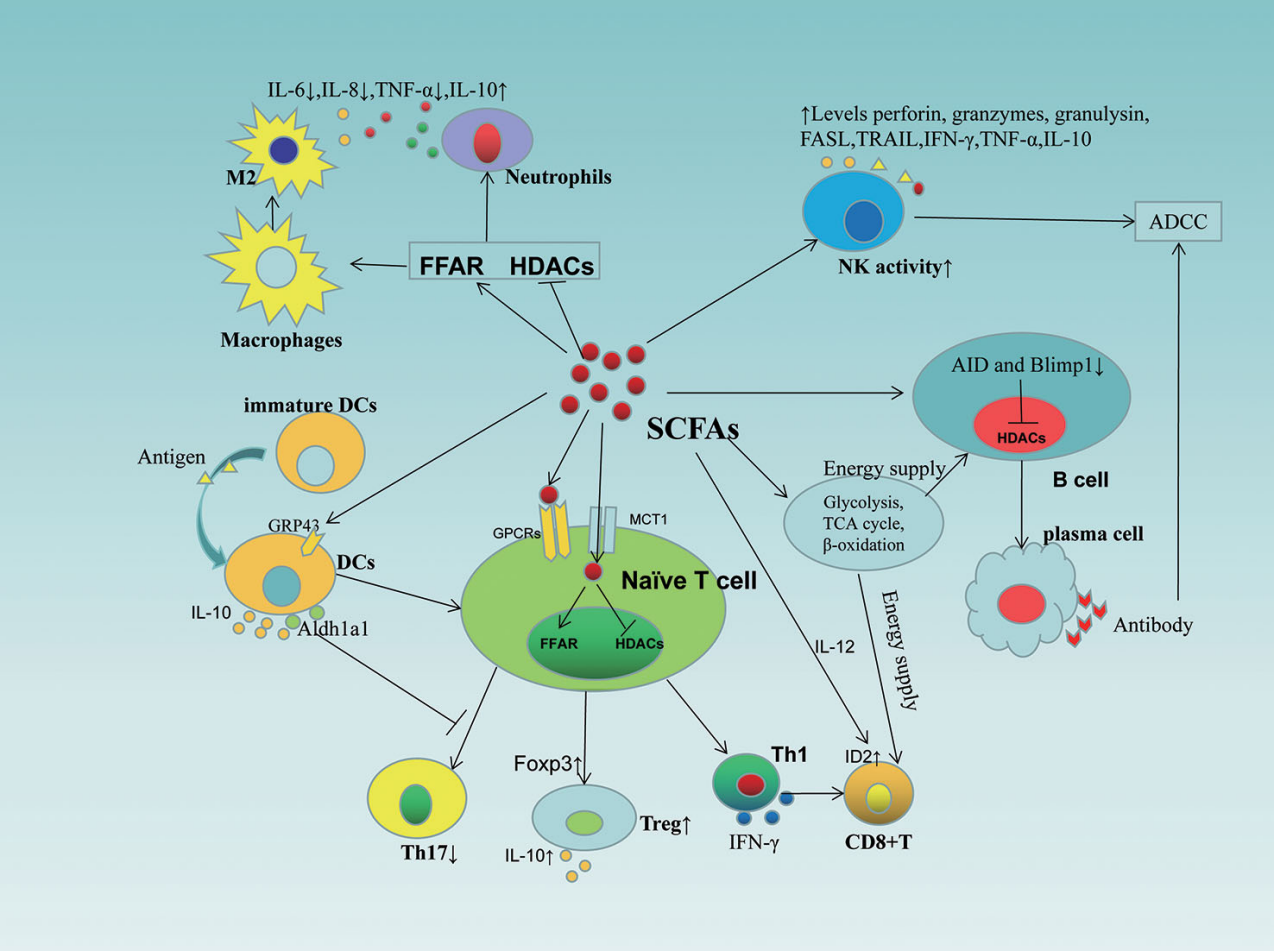
Role of SCFAs in immune cells. SCFAs affect the activity, differentiation and function of immune cells (including T cells, B cells, NK cells, DCs, macrophages, neutrophils) through different mechanisms. SCFAs, short-chain fatty acids; GPCRs, G-protein-coupled receptors; HDACs, inhibit histone deacetylases; FFAR, free fatty acid receptor; ADCC, antibody dependent cell-mediated cytotoxicity; IL, interleukins; MCT1, monocarboxylate transporters 1; FASL, Fas Ligand; TRAIL, tumour necrosis factor-related apoptosis-inducing ligand; NK, natural killer cells; DC, dendritic cell; Aldh1a1, aldehyde dehydrogenases 1 family member a1; AID, activation-induced cytidine deaminase.

Gut microbiota dysbiosis and microbiota-associated metabolites play a key role in tumorigenesis and immunotherapy. SCFAs are major metabolites produced by the fermentation of insoluble dietary fiber by intestinal microorganisms, which serve as energy substrates linking dietary patterns and gut microbiota (104) and play a key role in intestinal microbial immune and metabolic networks affecting the activity of immune and tumor cells (105)(**Figure 3**), with possible mechanisms including: 1) inhibition of HDAC activity and calcium-regulated phosphatase-mediated activation of nuclear factor of activated T cells 3 (NFATc3) transcription factor, thereby blocking tumor cell proliferation(106) and activating cell cycle inhibitor p21 *via* GPR43, downregulating inhibitor of apoptosis proteins (IAP) inhibitor, inhibiting cancer cell proliferation, and inducing apoptosis (107). 2) Inducing CD8^+^T cell ID2 expression *via* IL-12 signaling thereby directly enhancing the antitumor cytotoxicity of CD8^+^T, while promoting the expression of effector molecules such as IFN-γ and TNF-α and enhancing the antitumor effects of cytotoxic T lymphocytes (CTL) (108). 3) SCFA provides energy to B cells, memory T cells and effector T cells by regulating metabolic pathways (e.g. glycolysis, TCA cycle and β-oxidation) to enhance the efficacy of immune checkpoint inhibitors (ICI) (109–111). And that changes in nutrient composition can modulate the antitumor effects of SCFA by affecting gut microbes. For example, the WD, on the one hand, slows down mucus growth and increases the permeability of the colonic mucus barrier, leading to a shift in intestinal flora and causing changes in the metabolic and immune system, and on the other hand, the intestinal microbial community changes, with a gradual decrease in SCFA-producing bacteria (e.g., *Bifidobacteria*) and an increase in *Firmicutes phylum* (112), resulting in a decrease in SCFA secretion, leading to a disruption of intestinal immune homeostasis and promoting inflammation as well as the expression of cancer-related genes (101). Fiber intake increases the number of bacteria (*Bifidobacterium and Lactobacillus*) that produce SCFAs (113), a change that increases SCFA production, inhibits HDACi, enhances the anti-inflammatory phenotype in colonic macrophages and dendritic cells (DC) by activating GPR109a, and regulates regulatory T cell (Tregs) function to suppress inflammation and maintain intestinal immune homeostasis (101), thereby reducing cancer risk. Marine omega-3 fatty acid intake resulted in increased abundance of *Lactobacillus* and *Bifidobacterium* and decreased *F. nucleatum* and LPS-producing bacteria (114), contributing to the maintenance of intestinal barrier integrity, reduced oxidative stress and decreased inflammation. In addition, SCFA-producing bacteria, such as *Bifidobacterium* and *Faecalibacterium*, promote antitumor immunity and facilitate cancer immunotherapy, making patients more sensitive to immunotherapy responses (115, 116). However, the level of SCFAs seems to be very important in inducing its function. Evidence shows that the anti-inflammatory effect of SCFAs is dose-dependent (117). Qi Hui et al. (118) found that when butyrate concentration is 20 μmol, the levels of IL-6 and IL-1βwere significantly reduced, while the concentration was 2 μmol has no effect. Coutzac et al. (119) also found that Tregs increased at a butyrate concentration of 50 μmol or 100 μmol, but not at a concentration of 10 μmol. The effect of SCFAs on Th1/Th17 cells only occurs at relatively high concentrations (120). When SCFAs increases *in vivo* for a long time, which is higher than the physiological level, it will cause T cell reaction disorder, produce Th1 and Th17 cells, and thus induce tissue inflammation (121).

Inosine, a purine metabolite of *Akkermansia muciniphila* and *Bifidobacterium pseudolongum*, plays an important role in improving the efficacy of ICI (122). On the one hand, inosine enhances the ability of tumor cells to present tumor antigens, thus enabling cytotoxic immune cells to readily recognize and kill tumor cells (105). On the other hand, it stimulates phosphorylation of cAMP response element binding protein (pCREB) through the inosine-A2AR-cAMP-PKA signaling pathway, upregulates IL-12Rβ2 and IFN-γ transcription, and promotes the differentiation and accumulation of Th1 cells in the tumor microenvironment (TME), thus enhancing the potency of ICI (122). Furthermore, when glucose is restricted, inosine serves as an alternative carbon source for CD8^+^T cells and alleviates the restriction of CD8^+^T lymphocyte energy metabolism in tumor cells (123).

LPS is a major component of the outer membrane of Gram-negative bacteria and is recognized by the host immune system, and the release of large amounts of LPS causes an uncontrolled immune response (124). Animal experiment (125) showed that LPS-mediated chronic inflammation induced immunosuppressive microenvironment enhanced tumorigenesis. Meanwhile, LPS enhanced the invasive potential of cancer cells by activating the stromal cell-derived factor 1α (SDF-1α)/CXCR4 axis and the onset of epithelial-mesenchymal transition (EMT) through the NF-κB signaling pathway, thus contributing to enhanced cell migration and invasion (126). In addition, the study (127) found that LPS was abundant in CRC tissues and was associated with a low response to anti-PD-L1 mAb treatment. However, LPS has also been shown to be an effective immune booster with potential anti-tumor capabilities. Previous animal trials (128) observed that intravenous LPS completely abrogated immunogenic tumors, but subsequent phase I/II clinical trials conducted were not as effective (129, 130). Further studies have shown that a change in the route of administration (e.g., intratumoral injection) may be more effective in treating cancer and can lead to tumor regression (131). Stronger protective anti-tumor immunity was observed in mice receiving LPS pretreated tumor cells, which was achieved by activating antigen-specific CD8+ T cell responses and reducing MDSCs (132). The use of LPS to activate strong pro-inflammatory responses induced by TLRs to break tumor-associated immune tolerance is also one of the mechanisms to enhance antitumor responses (133). LPS, as an agonist of TLR4, is able to activate anticancer immune responses through the TLR4 pathway, including induction of maturation and migration of DCs, activation of macrophages, B cells, NK cells, and cytotoxic T cells (133).

In addition to SCFA,inosine and LPS, other metabolites affect the antitumor immune response, including anacardic acid and Bile acid (105). The metabolite anacardic acid of Bacteroides caccae induces macrophage activation through phosphorylation of ERK1/2, JNK, P38 kinase and NF-κB, which activates innate immunity (134) and induces the production of neutrophil extracellular traps (NET), promoting the production of tumor-infiltrating immune cells by macrophages, NK cells and T lymphocytes to regulate adaptive and antitumor immunity (135, 136). Bile acid inhibits Th17 cell differentiation (137), attenuates immunostimulatory properties when acting on DCs, thereby inducing Foxp3 expression, upregulating Treg numbers, and promoting immune escape (138), to the detriment of antitumor immunity. However, paradoxically, it has been shown that gut microbiota-mediated bile acid metabolism increases the abundance of CXCR6^+^NKT cells in the liver and exerts antitumor effects in hepatocellular carcinoma (139). This suggests that there are differences in the roles played by gut microbial metabolites in different scenarios, and continuing to explore the role of these metabolites in different cancers will be one of the future research directions.

Another potential mechanism for this is the reprogramming of TME immunity by the gut microbiota through binding to innate and/or adaptive immune cells (105). For instance, *Bifidobacterium bifidum* increases DC activation, thereby improving tumour-specific CD8^+^T-cell responses and restoring anti-PD-L1 therapeutic efficacy (140). Concurrently, CD47-based immunotherapy is promoted in a stimulator of interferon genes (STING) signaling and IFN-I-dependent manner (141). *Baeteroides fragilis* enhances the antitumor effects of CTLA-4 blockade by triggering DC maturation and stimulating IL-12-dependent Th1 cell immune responses (142). Besides, *Baeteroides fragilis* induces macrophage phenotypic polarization to M1 and upregulates cellular expression of CD80 and CD86, promoting innate immunity (143). *Lactobacillus* plantarum effectively increases the expression of natural cytotoxicity receptor (NCR) proteins and promotes NK cell activation to trigger innate immunity (144). These regulatory effects of gut microbes are modulated by dietary factors. It was found that a high-salt diet increased intestinal permeability and intra-tumoral *Bifidobacterium* localization, which enhanced NK cell activation to induce anti-tumour immunity (145). High-fiber diet-induced microbial alterations derived from STING agonists induced IFN-I signaling *via* intra-tumour monocytes, shifted mononuclear phagocytes to anti-tumour macrophages (Macs) and triggered monocyte-IFN-I-NK-DC crosstalk, which enhanced anti-tumour responses and ICI efficacy (146).

Earlier studies have shown that the gut microbiota can stimulate anti-tumor immune responses by regulating CD8^+^T cells (147), Th1 (148) and tumor-associated myeloid cells (149). *Bifidobacterium, Enterococcus, faecalibacterium, Ruminococcus* and *Clostridium* promote CD8^+^ T lymphocyte infiltration in tumor tissues. *Firmicutes* and *Actinobacteria* enhance the activation of CD56^+^CD8^+^ T cells in peripheral blood of ICI responders (150, 151). *Bifidobacterium pseudolongum* and *Bacteroides fragilis* stimulated Th1 immune response to enhance ICI efficacy (122, 142). *Faecaliberium* increased the proportion of CD4^+^ T cells and decreased the proportion of Treg cells in peripheral blood (152). In addition, a study has isolated a consortium of 11 bacterial strains from healthy human donor feces that were able to induce an increased ratio of IFNγ^+^CD8^+^T cells in a manner dependent on CD103^+^DCs and major histocompatibility class (MHC) Ia molecules, enhancing the therapeutic effect of immune checkpoint inhibitors (153).

In addition, gut microbes play an important role in enhancing immunogenicity. On the one hand, gut microbiota can enhance ICI responses by acting on UBA6 on the surface of tumor cells, thus directly enhancing the innate immunogenicity of tumor cells. On the other hand, the gut microbiota can promote the efficacy of ICI by providing tumor cross-antigens to indirectly increase the immunogenicity of tumor cells (153–155). The effect of intestinal microorganisms on cancer immunotherapy is under further study (**Table 2**).

**Table 2:** Clinical trial of the influence of intestinal microorganisms on cancer immunotherapy.

Identifier	Title	Trial Subject	Number	Trial type	End time
NCT05065515	Establishment of Individualized Immunotherapy Strategy and Platform Based on Changes of Intestinal Microbiota	upper gastrointestinal cancer	40	Observational	2023-12
NCT04957511	Gut Microbiome and Treatment for Gynecological Cancer Patients Receiving Immunotherapy	Gynecological Cancer	30	Observational	2023-06
NCT05199649	Correlation Between the Efficacy of Neoadjuvant Chemotherapy Combined With Immunotherapy of Operable Thoracic Esophageal Squamous Cell Carcinoma and the Metabolites of Intestinal Flora	Esophageal Squamous Cell Carcinoma	30	Observational	2023-06-01
NCT04682327	Gut Microbiota and Cancer Immunotherapy Response	NSCLC	50	Observational	2022-12-30
NCT05008861	Gut Microbiota Reconstruction for NSCLC Immunotherapy	NSCLC	20	Interventional	2022-12-30
NCT03353402	Fecal Microbiota Transplantation (FMT) in Metastatic Melanoma Patients Who Failed Immunotherapy	Metastatic Melanoma	40	Interventional	2021-12-30
NCT03643289	Predicting Response to Immunotherapy for Melanoma With Gut Microbiome and Metabolomics (PRIMM)	Melanoma	450	Observational	2023-05-02
NCT05083416	Effect of Prolonged Nightly Fasting on Immunotherapy Outcomes in HNSCC - Role of Gut Microbiome	Head and Neck Cancer	52	Interventional	2024-08
NCT04566029	Evolution of Proteomic Profiles of Intestinal Microbiota in Patients With Locally Advanced or Metastatic Urothelial Carcinomas (AMI)	Urothelial Carcinomas	40	Observational	2023-06-01
NCT03891979	Gut Microbiome Modulation to Enable Efficacy of Checkpoint-based Immunotherapy in Pancreatic Adenocarcinoma	Pancreatic Adenocarcinoma	–	Interventional	2020-06-01
NCT05462496	Modulation of the Gut Microbiome With Pembrolizumab Following Chemotherapy in Resectable Pancreatic Cancer	Pancreatic Cancer	25	Interventional	2029-04
NCT02960282	Gut Microbiome in Fecal Samples From Patients With Metastatic Cancer Undergoing Chemotherapy or Immunotherapy	Metastatic Cancer	21	Observational	2022-04-13
NCT04638751	ARGONAUT: Stool and Blood Sample Bank for Cancer Patients	NSCLC, TNBC, CRC, pancreatic cancer.	4000	Observational	2024-12
NCT04291755	Development and Analysis of a Stool Bank for Cancer Patients	NSCLC,CRC	100	Observational	2022-12-31

We already know that there is a strong link between diet, gut microbial composition and the outcome of immunotherapy (23). In view of this, many dietary therapies based on gut microbiota have emerged for the treatment of oncology patients. In recent years, attention has been paid to dietary therapies for tumor patients, i.e., the popular “ cancer specific “ diets. One of the mechanisms underlying these dietary patterns is alteration of the gut microbiota (156). Fasting is a dieting pattern that animal models confirm to be beneficial for some types of cancer, and one of its major mechanisms is mediated by gut microbes. Such as every-other-day fasting (EODF) leads to increased levels of *Firmicutes* and increased SCFAs production, while also reducing the abundance of potentially pathogenic *Proteobacteria* and concomitantly increasing *Akkermansia muciniphila* levels (157, 158). A diet low in carbohydrates and rich in fiber, which reduces the growth of pro-inflammatory microorganisms such as *Bacteroides acidifaciens*, *Escherichia coli*, *Ruminococcus gnavus*, and *Clostridium cocleatum*, increases the growth of anti-inflammatory microorganisms such as *Lachnospiraceae* (159). Recent studies have shown that higher microbial alpha diversity and abundance of the *Ruminococcaceae* family and the genus *Faecalibacterium* in patients with adequate dietary fiber intake enhanced the effect of immunotherapy, with patients having significantly longer Progression-free survival (PFS), and that the effect on treatment efficacy depended on the microbiota, fiber-fermenting *Ruminococcaceae* family of bacteria may promote fiber-fighting tumor immunity by influencing the pathways of T cell activation and T cell aggregation in tumors, including inducible T cell costimulator (ICOS)-expressing CD8^+^ and CD4^+^ T cells (160). The KD can lead to enhanced anticancer immune response by inhibiting lactic acid-mediated tumor immunosuppression and myeloid-derived suppressor cells (MDSC) expression in tumor-bearing mice (161) and has significant efficacy in tumor chemotherapy and radiotherapy (162), but the effect on immunotherapy remains to be determined (163). In addition, some nutrients can influence multiple mechanisms such as cell signaling, apoptosis and immune system regulation, and strongly regulate the composition of the gut microbiota, which plays a key role in maintaining gut homeostasis and is associated with the regulation of host inflammatory and immune responses (164, 165). From this point on, the diet-gut microbiota axis has a potential role in tumorigenesis, immunomodulation and therapy, and perhaps intervention with nutrition and microbes based on standard antineoplastic therapies may have better efficacy.

There are many dietary strategies available to modulate the composition or metabolic/immune activity of the human gut microbiota to enhance the antitumor immune response (166). Supplementation of some micronutrients and vitamins also favors anticancer immune responses, and their effects on immune function have been summarized in detail (REF (167)). Probiotics, prebiotics and polyphenols are the most established (**Table 3**). Their use is also considered as a potential anti-cancer alternative or adjuvant therapy (168). Polyphenolic compounds, which are widely found in fruits, vegetables and grains, have potential benefits for human health. Dietary polyphenols exert probiotic-like effects that help maintain a healthy gut and reduce inflammation levels by stimulating the growth of beneficial bacteria and inhibiting the development of pathogenic microorganisms (169, 170), with a potential role in the treatment of cancer. For example, resveratrol could enhance the anti-cancer immunity of tumor-bearing mice by promoting the accumulation of effector CD8^+^T cells and monocyte MDSCs and suppressing the number of tumor-derived CD4^+^CD25^+^Tregs and CD8^+^T cells suppressor granulocyte MDSCs (171, 172). Curcumin contributes to the treatment of inflammatory bowel disease by inhibiting NF-κB activation and inducing mucosal Treg cells and inducing changes in intestinal microbial structure and fecal SCFAs levels (173), which has potential therapeutic effects in some inflammatory tumors. To date, no trials have reported the efficacy of polyphenols on immunotherapy, but polyphenols have immunomodulatory activity and can counteract inflammatory processes in the tumor microenvironment by regulating the production of cytokines and chemokines in turn (174), making them an effective adjunct to future cancer immunotherapy.

**Table 3:** Clinical trial of the effect of registered diet strategy on cancer immunotherapy.

Identifier	Title	Diet	Drugs	Number	Trial type	End time
NCT04645680	Effect of Diet on the Immune System in Patients With Stage III-IV Melanoma Receiving Immunotherapy, DIET Study (DIET)	Whole foods diet	Pembrolizumab/nivolumab	42	Interventional	2023-02-01
NCT04866810	The Effect of Diet and Exercise on ImmuNotherapy and the Microbiome (EDEN)	plant-based, high-fiber diet	relatlimab and nivolumab	80	Interventional	2025-10-31
NCT05356182	A Pilot and Feasibility Study of a Dietary Intervention With Low-protein Meals in Cancer Patients Receiving Immunotherapies	low-protein diet	ICI (specific unknown)	30	Interventional	2024-04
NCT03700437	Fasting-mimicking Diet With Chemo-immunotherapy in Non-small Cell Lung Cancer (NSCLC)	Fasting-mimicking Diet	carboplatin/pemetrexed and pembrolizumab	12	Interventional	2022-03-22
NCT04316520	Ketogenic Diet for Patients Receiving First Line Treatment for Metastatic Renal Cell Carcinoma (CETOREIN)	Ketogenic Diet	nivolumab+ipilimumab, pembrolizumab+axitinib, sunitinib or pazopanib.	20	Interventional	2024-05
NCT05220124	A Study of Probiotics Administration in the Immunotherapy of Urothelial Bladder Carcinoma	Live Combined (*Bifidobacterium,Lactobacillus* and *Enterococcus Capsules*)	Not Applicable	190	Interventional	2024-11-30
NCT04699721	Clinical Study of Neoadjuvant Chemotherapy and Immunotherapy Combined With Probiotics in Patients With Potential/​Resectable NSCLC	Prebiotic	nivolumab	40	Interventional	2027-12
NCT05083416	Effect of Prolonged Nightly Fasting on Immunotherapy Outcomes in HNSCC - Role of Gut Microbiome	Prolonged Nightly Fasting (PNF)	Nivolumab,pembrolizumab, Atezolizumab, Avelumab or Durvalumab	52	Interventional	2024-08

The combination of cancer immunotherapy with probiotics is recognized by many researchers because probiotics not only complement immunotherapy, but can improve the safety and reduce the side effects of cancer treatment (175), thanks to the ability of probiotics to alleviate dysbiosis and improve anti-cancer immunity (163). For example, *Lactobacillus rhamnosus* GG can inhibit the expression of inflammatory proteins NF-κB-p65, COX-2 and TNF-α (176). *Lactobacillus plantarum* prolongs survival of tumor-bearing mice by enhancing effector CD8^+^T cell function, CD4^+^T cell differentiation, and NK cell intratumoral infiltration (177). The combination of *Lactobacillus acidophilus* NCFM and *Bifidobacterium lactis* BI-04 resulted in enrichment of butyrate-producing bacteria (e.g., *Clostridium* and *Fasciola*) in the colonic mucosa, as well as a reduction in CRC-associated genera, including *Fusobacterium* and *Peptostreptococcus* (178).

Animal assays (179) showed that *Lactobacillus acidophilus* cell lysates enhanced the antitumor activity of anti-CTL antigen-4 blocking antibody (CTLA-4 mAb) in model mice, which was attributed to an increase in CD8^+^ T cells, an increase in effector memory T cells (CD44^+^CD8^+^CD62L^+^), and a decrease in Treg (CD4^+^CD25^+^Foxp3^+^) and M2 macrophages (F4/80^+^CD206^+^) in the tumor microenvironment, along with a partial restoration of CRC-associated dysbiosis. Previous studies have also reported that *Bifidobacterium bifidum* promotes antitumor responses by enhancing dendritic cell function and promoting T cell-mediated metabolism (140, 180). Supplementation of *E. coli Nissle* 1917 to tumor-bearing mice also improved the efficacy of galunisertib by enhancing tumor-specific effector T cell infiltration and dendritic cell activation to alleviate the immunosuppressive tumor microenvironment, thereby enhancing the therapeutic effects of galunisertib for tumor growth inhibition and metastasis suppression (181). Recently, Shi et al. (182) reported that oral administration of *Akkermansia muciniphila* (AKK) to tumor-bearing mice significantly enhanced the therapeutic efficacy of IL-2-based immunotherapy. The mechanism may be through activation of Amuc, an outer membrane protein of toll-like receptor 2 (TLR2) signaling, resulting in effective tumor regression. In addition to the role of probiotics themselves, probiotic derivatives also contribute to tumor immunotherapy. Kawanabe-Matsuda et al. (183) reported that microbial exopolysaccharide produced by *Lactobacillus delbrueckii* subsp. bulgaricus OLL1073R-1 (EPS-R1) induced an increase in CCR6^+^ CD8^+^T cells and production of IFN-γ in Peyer’s patches, accompanied by the expression of a large number of immune response genes, maintained T cell function, and improved the tumor microenvironment, enhancing the antitumor effects of anti-CTLA-4 or anti-PD-1 monoclonal antibodies against CCL20-expressing tumors. However, other studies have yielded conflicting results, with some preclinical models finding reduced frequency of cytotoxic T cells, increased tumorigenesis, and impaired antitumor immunity in the tumor microenvironment of probiotic-treated mice (160, 184). Therefore, the effects of different probiotics on immunity and cancer immunotherapy responses need to be more carefully investigated.

In addition, the recent developed fecal microbiota transplantation (FMT) as an effective way to modulate gut microbiota composition and diversity has been shown to improve the response to ICIs (185). FMT of cancer patients responding to ICIs into sterile or antibiotic-treated mice improved the antitumor effects of PD-1 blockers (186) (29097494). In melanoma patients, gut microbial diversity was richer in ICI-responding patients compared to non-responders. After transplantation of responder colonies in germ-free recipient mice, response to PD-L1 treatment improved, and further analysis revealed higher densities of peripheral CD4+T and CD8+ T cells, upregulation of PD-L1 in the tumor microenvironment, significant enrichment of innate effector cells, and suppressor myeloid cells was reduced (115). In another study in metastatic melanoma, transplantation of feces from responding patients into germ-free mice was shown to improve tumor control, enhance T-cell response, and improve the efficacy of anti-PD-L1 therapy (116). Two recent clinical trials (150, 151) demonstrated that FMT of ICI responder origin altered the gut microbiome of non-responders and reprogrammed TME to overcome resistance to PD-1 blockade. In conclusion, FMT modulates gut microbes in dialogue with the immune system and could be a potential approach for immunotherapy of several cancers.

## Conclusions

Diet, as one of the key influences on gut microbes, plays an important role in the composition of gut microbes, depending on the nutritional composition of different dietary patterns. Diet interacts with gut microbes and they are involved in cancer development and treatment by influencing processes such as host metabolism and immunity. There is established evidence that dietary strategies based on the crosstalk between diet and gut microbes have a potentially beneficial role in cancer immunotherapy. However, the information we currently have is insufficient to fully determine how different diets affect the effectiveness of immunotherapy, and more detailed studies are needed to examine how different diets affect certain bacterial species in the gut microbiota to better determine their impact on the effectiveness of immunotherapy (**Table 4**). Undoubtedly, dietary interventions based on diet-gut microbiota interactions will be an exciting prospect for application.

**Table 4:** Immune effects of some dietary supplementation.

Category	Immune action
Prebiotics	Influencing immune modulation by exploiting the metabolic activities of commensal intestinal bacteria; Inhibit adhesion molecule expression, chemokine formation, monocyte/macrophage immune activity and neutrophil recruitment; Promotes upregulation of Tregs; Inhibit the release of proinflammatory mediators and promotes anti-inflammatory mediator secretion (levels IL-10↑, PGE2↑,SOD1↑,IL-1↓,IL-6↓,TNF-α↓,NO↓, NF-kB↓)
Probiotics	Restore innate and adaptive immunity; Promote differentiation of T cells to Treg cells and Th2 phenotype; Anti-inflammatory activity; Enhanc mucosal immune defenses, amplified immunoglobulin A production and cytokine stimuli(levels IL-10↑, IL-1↓,IL-6↓,TNF-α↓);Stimulate GALT, MLNs, ILFs, TLRs, expression of defensins, lectins and other antimicrobial proteins.
Postbiotics	Improve intestinal barrier function; Induce the expression of defensins; Immune stimulation activity; Enhance the cascade of innate and adaptive immune response of host; Anti-inflammatory activity(IL-1β↓, IL-6↓, IL-12↑, TNF-α↓, IL-10↑)
Polyphenols	Immunomodulatory activity; Regulate the production of cytokines and chemokines; Induce Tregs; Anti-inflammatory activity; Promote the accumulation of effector CD8+T cells; Suppress tumor-derived CD4+CD25+Tregs.
Synbiotics	Increased *Lactobacillus* and *Bifidobacterium* genus count and maintenance of balance of the intestinal microbiota; Improved immunomodulative abilities; Inhibit the production of pro-inflammatory cytokines (e.g. TNF-α); Increase intestinal IgA level.

PGE2, prostaglandin E2; SOD1, superoxide dismutase 1; IL, interleukin; GALT, gut-associated lymphoid tissues; MLNs, metastatic lymph nodes; ILFs, isolated lymphoid follicles; TLRs, toll-like receptors; TNF, tumor necrosis factor.

## Author contributions

Conceptualization, XW; writing—original draft preparation, XW and SG; writing—review and editing, XW and SG. All authors contributed to the article and approved the submitted version.
